# Supplementary material for: Improving Learning and Study Strategies in Undergraduate Medical Students: A Pre-Post Study
Source: Healthcare (Basel). 2023 Jan 28;11(3):375. doi: 10.3390/healthcare11030375 (PMC9914150; doi:10.3390/healthcare11030375)

Improving Learning and STUDY Strategies in Undergraduate Medical Students: A Pre-Post Study

Supplementary Table S1. Comparison between study participants that completed the UKASP compared to those who lost to follow-up (LTFU)

Characteristic	No LTFU n=64	LTFU n=14	*p*-Value*
Age (yr), median (IQR)	21 (21-22)	21 (21-22)	0.86
Sex, n (%)			
Female	42 (65.6)	9 (64.3)	1
Marital status, n (%)			0.49
Single	47 (73.4)	12 (85.7)	
Relationship	17 (26.6)	2 (14.3)	
Married	0 (0)	0 (0)	
Occupational status, n (%)			1
Yes	4 (6.2)	1 (7.1)	
Living arrangements, n (%)			0.63
Alone	9 (14.1)	1 (7.1)	
Rommate	8 (12.5)	3 (21.4)	
Family	47 (73.4)	10 (71.4)	
Parenthood, n (%)			1
Yes	1 (1.6)	0 (0)	
Medical parents, n (%)			0.68
Yes	10 (15.6)	1 (7.1)	
High school attended, n (%)			1
Public	6 (9.4)	1 (7.1)	
Private secular	47 (73.4)	11 (78.6)	
Religious	8 (12.5)	2 (14.3)	
Municipal	3 (4.7)	0 (0)	
College degree before Med School, n (%)			0.45
Yes	2 (3.1)	1 (7.1)	
English level perception, n (%)			0.68
Basic	5 (7.8)	2 (14.3)	
Intermediate	29 (45.3)	5 (35.7)	
Avanced	30 (46.9)	7 (50)	
GPA medicine/4, median (IQR)	3.28 (3.14-3.46)	3.34 (3.18-3.61)	0.42
Pasteur scholarship, n (%)			1
Yes	6 (9.4)	1 (7.1)	
Financial assistantship, n (%)			0.85

Yes	32 (50)	8 (57.1)	

UKASP: "USFQ's Keys to Academic Success Program", IQR: Interquartile range
*Continuous variables were analyzed by Wilcoxon Rank Sum Test and categorical data were analyzed through Chi-squared or Fisher´s exact test as appropriate.

Supplementary Table S2. Comparison (median and IQR) between study participants with post-test scores vs. no post-test scores

Scale	Post-test Yes	Post-test No	*p*-Value*
ANX	35 (5-60)	10 (5-37.5)	<0.001
ATT	30 (10-50)	50 (27.5-60)	<0.001
CON	35 (15-60)	22.5 (10-41.3)	<0.001
INP	55 (35-80)	47.5 (35-76.3)	<0.001
MOT	45 (25-80)	40 (8.8-68.8)	<0.001
SFT	60 (25-75)	57.5 (12.5-85)	<0.001
SMI	35 (20-70)	35 (12.5-66.2)	<0.001
STA	30 (15-60)	32.5 (13.7-76.2)	<0.001
TMT	25 (10-45)	25 (5-65)	<0.001
TST	45 (20-60)	22.5 (7.8-56.2)	<0.001

ANX, Anxiety; ATT, Attitude; CON, Concentration; INP, Information Processing; MOT, Motiva-tion; SFT, Self-Testing; SMI, Selecting Main Ideas; STA, Study Aids; TMT, Time Management; TST, Test Strategies; IQR, Interquartile range. *P-values were calculated by using Wilcoxon Rank Sum Test.

Supplementary Table S3. Sensitivity analysis with attendees that attended three or more workshops (n=26)

Scale	Pre-test	Post-test	Percent change between pre-test and post-test*	*p*-Value†
ANX	25 (5-53.7)	52.5 (30-72.5)	110	0.02
ATT	17 (10-50)	50 (30-67.5)	194.1	<0.01
CON	25 (10-45)	50 (36.2-68.7)	100	<0.01
INP	55 (40-78.7)	75 (56.2-85)	36.4	0.4
MOT	32.5 (25-65)	60 (45-80)	84.6	0.04
SFT	52.5 (21.2-75)	67.5 (40-83.7)	28.6	0.2
SMI	37.5 (20-77.5)	52.5 (31.2-70)	40	0.04
STA	25 (15-58.7)	50 (26.2-78.7)	100	0.04
TMT	12.5 (10-35)	40 (35-65)	220	<0.001
TST	32.5 (16.2-70)	70 (40-78.7)	115.4	<0.01

*Percent changes correspond to (Post-test value – Pre-test value)/ Pre-test value)*100
†p-value calculated by Sign test

Supplementary Table S4. Sensitivity analysis with attendees that attended four or more workshops (n=8)

Scale	Pre-test	Post-test	Percent change between pre-test and post-test*	*p*-Value†
ANX	35 (26.2-71.2)	60 (30-70)	71.4	0.73
ATT	20 (8.7-37.5)	40 (25-52.5)	100	0.28
CON	17.5 (5-31.2)	47.5 (35-71.2)	171.4	0.07
INP	50 (32.5-56.2)	67.5 (55-77.5)	35	0.29
MOT	27.5 (18.7-46.2)	60 (41.2-72.5)	118.2	0.07
SFT	37.5 (21.2-60)	52.5 (38.7-70)	40	0.29
SMI	25 (18.7-43.7)	42.5 (35-70)	70	0.07
STA	25 (18.7-32.5)	47.5 (36.2-61.2)	90	0.29
TMT	10 (1-12.5)	40 (28.7-55)	300	<0.01
TST	17.5 (13.7-36.2)	65 (43.7-71.2)	271.4	0.07

*Percent changes correspond to (Post-test value – Pre-test value)/ Pre-test value)*100
†p-value calculated by Exact Binomial Sign test due to the small sample size (<20)

Supplementary Figure S1. Timeline of the UKASP research project execution during the academic year 2019-2020

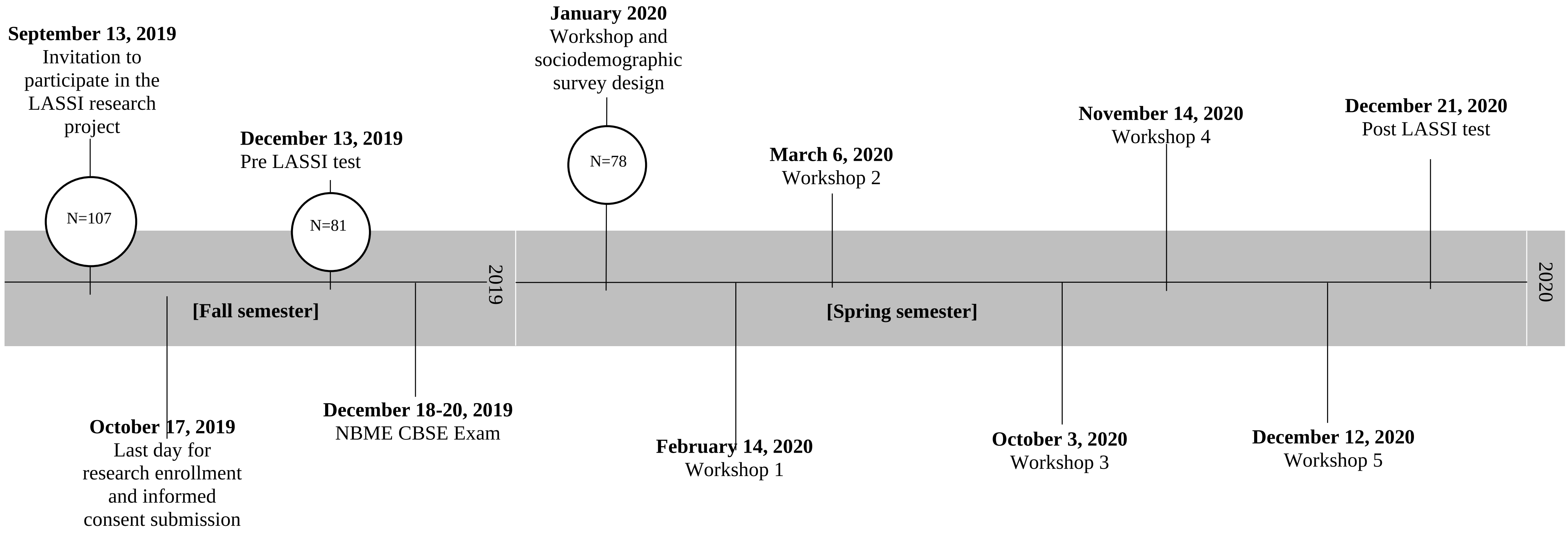

Supplement: Supplementary file 1 [file healthcare-11-00375-s001.zip › healthcare-2018229-supplementary.pdf]
